# Risk factors for development of acute kidney injury in hospitalised adults in Zimbabwe

**DOI:** 10.1371/journal.pone.0241229

**Published:** 2020-10-26

**Authors:** Alexander Gilbert, Lindsey Robertson, Jack E. Heron, Steve Chadban, Chiratidzo Ndhlovu, Rumbi F. Dahwa, David M. Gracey

**Affiliations:** 1 Department of Renal Medicine, Royal Prince Alfred Hospital, Camperdown, New South Wales, Australia; 2 Department of Medicine, University of Zimbabwe College of Health Sciences, Harare, Zimbabwe; 3 Kidney Node, Charles Perkins Centre, University of Sydney, Sydney, New South Wales, Australia; 4 Central Clinical School, Faculty of Medicine, University of Sydney, Sydney, New South Wales, Australia; University of Liège, BELGIUM

## Abstract

**Background:**

Acute kidney injury (AKI) is predominantly a disease of low and middle-income countries. Despite this, there is a particular paucity of data regarding AKI in Africa. Most published studies were conducted prior to the most recent Kidney Disease: Improving Global Outcomes (KDIGO) definition of AKI. This prospective, observational, cohort study examines AKI amongst newly admitted acute medical inpatients in a large, urban, tertiary hospital in Harare, Zimbabwe.

**Methods:**

All newly admitted, adult, medical patients in separate, randomly selected, 24-hour periods were included. Baseline demographic information, comorbidities, nephrotoxic medication use, and reason for admission were recorded on a standardised data capture record. A serum creatinine measurement was performed on all patients at the time of admission and again after 48 hours. Estimated glomerular filtration rate was calculated using the Chronic Kidney Disease Epidemiology Collaboration (CKD-EPI) equation and AKI was defined using the most recent KDIGO definition as an increase in the serum creatinine of greater than 26.5μmol/L within 48 hours, with admission creatinine used as a baseline measurement.

**Results:**

253 patients were included in the analysis; 137 patients (54.2%) were female; 100 patients (39.5%) had HIV infection. 36 patients (14.2%) met the KDIGO criteria for AKI during the 48-hour follow-up period. AKI was more common among males (19.8% vs 9.5%; p = 0.019). The AKI group had a higher serum creatinine at presentation than those without AKI (296.5μmol/L vs 91.0μmol/L; p<0.001) and at 48 hours (447.7μmol/L vs 77.1μmol/L; p<0.001). In logistic regression, AKI was related negatively to female sex (OR 0.461, 95% CI 0.211, 1.003; p = 0.051) and positively predicted by the presence of comorbid hypertension (OR 3.292, 95% CI 1.52, 7.128; p = 0.003) and chronic kidney disease (OR 6.034, 95% 1.792, 20.313; p = 0.004).

**Conclusions:**

KDIGO-defined AKI was common in hospitalised patients in Sub-Saharan Africa and was predicted by male sex, a history of comorbid hypertension and a history of comorbid chronic kidney disease.

## Introduction

Acute kidney injury (AKI) is predominantly a disease of low- and middle-income countries; although data in this setting are sparse compared to high-income countries [1]. There is a particular paucity of rigorous data from Africa and South East Asia [1]. As well, many studies in Africa were undertaken prior to the most recent Kidney Disease: Improving Global Outcomes (KDIGO) definition of AKI, resulting in inconsistent findings and an inability to compare outcomes [2, 3]. Rates of community-acquired AKI are difficult to define, particularly in settings where renal function is rarely measured and undiagnosed chronic kidney disease is common [4, 5]. The geographic distribution and burden of AKI in Africa, including its long-term morbidity and mortality, remain to be precisely defined, but are likely significant [6]. Creatinine measurement and calculation of the estimated glomerular filtration rate (eGFR) is often unavailable [7]; registry data are limited or not recorded, and studies investigating the long term follow-up of AKI are lacking [1, 8].

The characteristics of AKI amongst low and middle-income countries are different to those seen in high income countries, with more cases being community acquired and seen in younger patients from a single aetiology [3, 9, 10]. The aetiology of AKI in sub-Saharan Africa also varies geographically and seasonally, and include acute tubular necrosis, dehydration and overwhelming sepsis from fulminant infections, including malaria, tuberculosis, gastroenteritis and from opportunistic infections seen in people living with HIV (PLWHIV), amongst others [10–14]. Rapid urbanisation and an increase in the prevalence of non-infectious comorbidities, such as hypertension and diabetes, have also influenced the clinical characteristics of AKI in this setting [15].

Poor access to healthcare, delayed presentation and limited access to dialysis all contribute to the high mortality rate associated with AKI seen in this setting [15]. In one systematic review, only 33% of adults and 64% of children received dialysis when required [15]. The presence of AKI in Africa is associated with significant mortality [3, 14, 16] and an increased risk of developing chronic kidney disease (CKD) [10, 17].

There have been no published studies of AKI in Zimbabwe. We undertook the current study using the rigorous KDIGO-definition of AKI to examine the prevalence of AKI amongst inpatients on a medical ward in a large urban tertiary referral hospital and examine risk factors for AKI in this population.

## Methods

### Design, setting, population

This was a prospective, observational, cohort study of adult admissions to the general medical wards between July 2018 and February 2019 at the Parirenyatwa Group of Hospitals in Harare, Zimbabwe. The Parirenyatwa Group of Hospitals is the largest tertiary hospital centre in Zimbabwe, with a capacity of 1,800 beds. All patients over the age of 18 admitted to the medical wards in randomly chosen, separate, 24-hour periods were included. The timing of study enrolment was also dependent upon laboratory availability of reagents for the determination of the serum creatinine. Patients were screened for eligibility, and written informed consent was obtained. Known dialysis patients were excluded.

### Data collection

Baseline demographic information including age, sex and race, nephrotoxic medication use, as well as known co-morbidities including; hypertension, diabetes, hepatitis B, HIV infection, and CKD were recorded. Nephrotoxic medications recorded included non-steroidal anti-inflammatories (NSAIDs), antiretroviral therapy (ART), including tenofovir disoproxil fumarate (TDF), acyclovir and traditional medicines. The cause for the patient’s hospital admission was also sought. Data were collected from the medical record, and by interview with eligible patients by the same medical practitioner. These data were recorded on a pre-prepared data capture record. A serum creatinine was performed on all consenting patients at the time of admission, and at 48 hours. The serum creatinine was determined using the enzymatic method on a Beckman Coulter AU680 analyser; the results were isotope dilution mass spectrometry standardised. The eGFR was calculated using the Chronic Kidney Disease Epidemiology Collaboration (CKD-EPI) equation.

### Definitions

AKI was defined using the KDIGO AKI criteria, defining confirmed AKI as an increase in the serum creatinine of greater than 26.5μmol/L within 48 hours, using admission creatinine as a baseline measurement. The additional KDIGO criterion of a rise of 50–99% from baseline within 7 days was not used, given follow-up was limited to 48 hours [1]. None of the patients included in this study had a baseline serum creatinine value available from prior to their index admission.

Additionally, the stage of AKI was determined based upon KDIGO staging criteria. Stage 1 was defined as a 1.5–1.9 times rise in serum creatinine from admission, or a rise of 26.5μmol/L; stage 2 was a 2.0–2.9 times creatinine rise from admission; and stage 3 was a 3.0 times rise from admission [2]. The stage 3 criterion of a creatinine rise to greater than 353.6μmol/L was not utilised.

### Outcomes

The primary outcome of interest was the number of patients with AKI. Secondary outcomes examined in this study included the change in serum creatinine after 48 hours of hospital admission, and the presence of clinical risk factors, which may increase an individual’s risk of developing AKI.

### Statistical analysis

Continuous variables that were normally distributed are reported as mean, with standard error of the mean. Creatinine on admission and after 48 hours required log transformation; these variables are reported as back-transformed means and 95% confidence interval. A paired t-test was used to determine whether change in creatinine in the total group differed significantly from zero. One-way Analysis of Variance compared continuous variables in relation to AKI status. Independence between categorical variables was assessed using Fisher’s exact tests. Logistic regression was used to model predictors of AKI. A level of p<0.05 was considered significant. Statistical analysis was performed using Wizard: Statistics and Data Analysis Software for Mac, Version 1.9.40.

### Ethics

Ethical approval for this study was granted by the Department of Medicine and Clinical Director of the Parirenyatwa Group of Hospitals, the Joint Parirenyatwa University of Zimbabwe College of Health Sciences Research Ethics Committee and the Medical Research Council of Zimbabwe (MRCZ/B/1522).

## Results

Data was collected from 275 patients; 22 were excluded from the final analysis because of missing or incomplete data. This was primarily due to limited availability of creatinine reagent early in the study. 54.2% (n = 137) were female and the mean age was 48.4 years. All patients were of black African origin.

### Renal function

Table 1 demonstrates the renal function recorded at baseline and at 48 hours after admission for the group with AKI, those without AKI and for the total study population. The proportion with AKI in the total study population was 14.2% (n = 36). There were 27 patients (75.0%) with stage 1, 8 patients (22.2%) with stage 2 and 1 (2.8%) with stage 3 AKI. AKI was seen more commonly in males, compared to females (19.8% vs 9.5%; p = 0.019). The AKI group had a higher serum creatinine at presentation, compared to the group without AKI (296.5μmol/L vs 91.0μmol/L; p<0.001). The mean baseline creatinine for the total cohort was 99.1μmol/L. The patients with AKI had a higher serum creatinine at 48 hours after admission (447.7μmol/L vs 77.1μmol/L; p<0.001). By 48 hours the serum creatinine had increased by 120.7μmol/L in the AKI group, and had fallen by 21.4μmol/L in the group without AKI (p<0.001). The percentage change in serum creatinine observed in all patients was a fall of 8.6% (median -21.0%, IQR 8.9). In the group with AKI, the mean creatinine increase was 28.3% (median 13.7%, IQR 102.1). In the group without AKI, the mean change was a 11.6% decrease (median -23.6%, IQR 2.8). In the group of patients who had a fall in creatinine >26.5μmol/L, the mean percentage change in their creatinine was a 39.7% fall (median -50.7%, IQR -23.0). Of the patients included in our study, 93 (36.8%) presented with a raised admission serum creatinine (defined as >90μmol/L for females and >110μmol/L for males). Of the 44 females with an abnormal admission serum creatinine, we observed a mean decrease of 16.9% (median -43.8%, IQR 6.0). Of the 49 males with an abnormal serum creatinine, we observed a mean decrease in serum creatinine of 10.6% (median -26.0%, IQR 8.7).

**Table 1:** Renal function recorded at baseline and at 48 hours after admission for the group with AKI, those without AKI and for the total study population.

	Total N = 253	AKI absent N = 217	AKI present N = 36	P value between groups
Age	48.4 (SEM 1.1)	48.6 (SEM 1.3)	46.8 (SEM 2.6)	0.569
Sex, female (%)	137 (54.2)	123 (56.7)	13 (36.1)	0.019
Baseline eGFR mL/min	87.7 (SEM 3.0)	94.2 (SEM 3.0)	49.6 (SEM 8.8)	<0.001
Baseline creatinine μmol/L *	99.1 (CI 88.1, 111.4)	92.1 (CI 83.0, 100.0)	296.5 (CI 191.0, 460.3)	<0.001
48h creatinine μmol/L*	107.6 (CI 96.2, 122.2)	77.1 (CI 70.8, 84.1)	447.7 (CI 314.1, 636.8)	<0.001
Change in creatinine μmol/L	-2.9 (SEM 4.8)	-21.4 (SEM 3.5)	120.7 (SEM 14.1)	<0.001
Elevated admission creatinine (%)	93 (36.8)	69 (31.8)	24 (66.7)	<0.001
Chronic kidney disease (%)	14 (5.53)	6 (2.7)	8 (22.2)	<0.001

*Back transformed from log-transformed variable.

eGFR = estimated glomerular filtration rate, SEM = standard error of the mean

**Fig 1:**
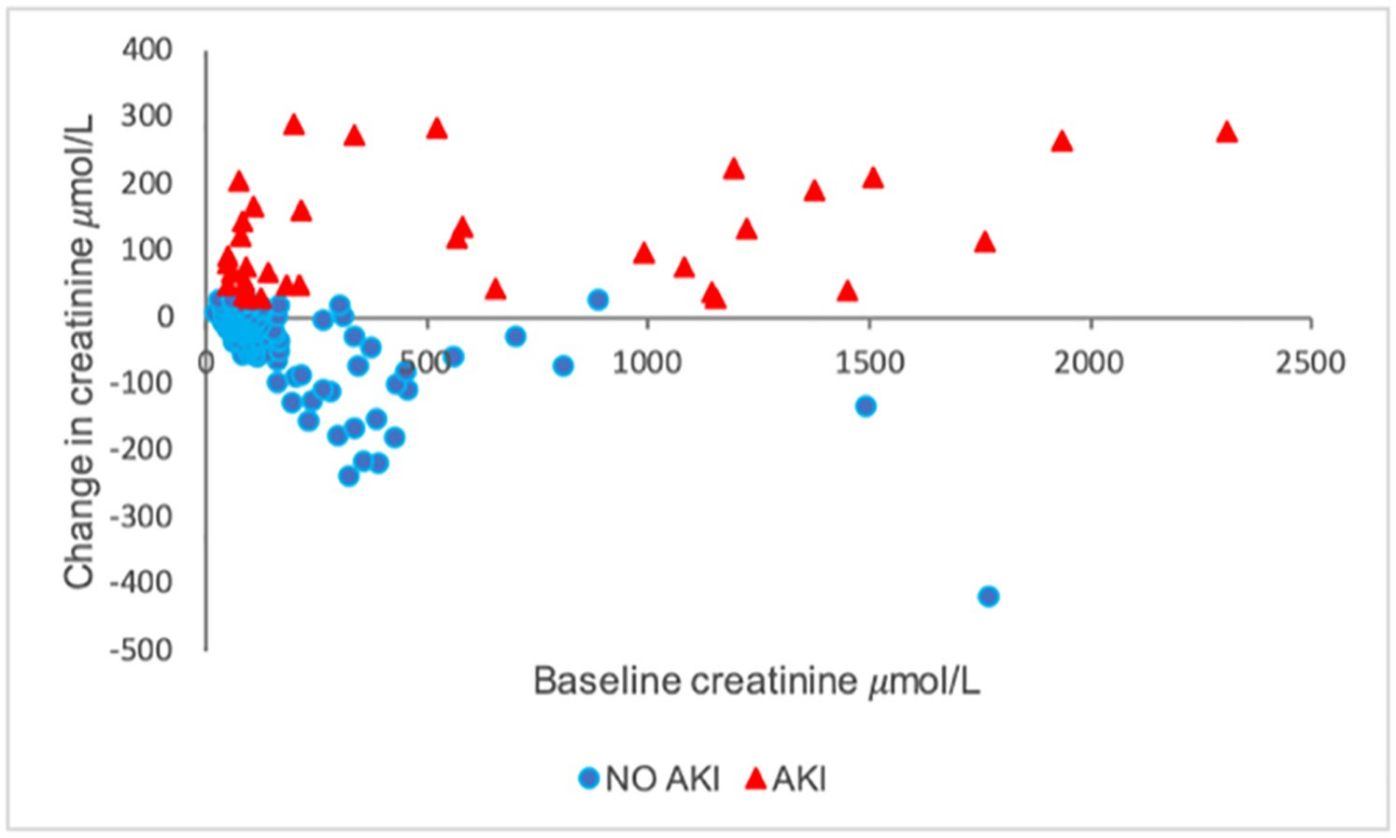
Change in serum creatinine in μmol/L by AKI group (AKI, or no AKI) observed at 48 hours, compared to baseline, for each patient.

The change in serum creatinine in the AKI group observed at 48 hours, compared to baseline, for each individual included in our study, is shown in Fig 1. Eleven patients (31%) in the AKI group had an admission serum creatinine of over 1000μmol/L, compared to 2 individuals (0.9%) in the no AKI group. The patients in the AKI group with an admission creatinine of over 1000μmol/L included 3 of the 14 patients with a past history of CKD; they were judged, clinically, to have acute-on-chronic renal dysfunction from a single, acute aetiology.

In our study population, 55 patients (22%) had a fall in their serum creatinine since their index admission of greater than 26.5μmol/L; of these individuals, 28 (51%) were male and 27 (49%) were female. Of these 55 individuals, 6 (11%) were known to have a prior history of CKD. Of those whose creatinine fell greater than 26.5μmol/L after 48 hours, 14 (25.5%) had an admission diagnosis of gastroenteritis, 7 (12.7%) were anaemic (defined as Hb <110g/L), and 23 (42%) were known to be infected with HIV. In a logistic regression analysis adjusted for baseline creatinine, an admission diagnosis of gastroenteritis and anaemia were significant predictors of a fall in creatinine of greater than 26.5μmol/L. A total of 91 patients (36%) included in our cohort had either an increase, or decrease, in their serum creatinine of greater than 26.5μmol/L during the observation period. Of the 14 patients with a known history of CKD included in our patient cohort, all had either an increase, or a decrease, in their serum creatinine of greater than 26.5μmol/L during this study.

### Clinical characteristics

Table 2 demonstrates the proportion of patients with AKI and the admission creatinine in those with and without certain clinical characteristics, including pre-existing comorbidities, and the reason for hospital admission.

**Table 2:** AKI and admission creatinine, by comorbidities and admission diagnoses.

Proportion with AKI (%)/ Admission creatinine (μmol/L)			
	Variable absent	Variable present	P value
**Co-morbidities**			
HIV N = 100 (39.5%)	28/153 (18.3%)	8/100 (8.0%)	0.022
	115.6 (99.3, 134.6)	96.6 (81.8, 114.3)	
Hypertension N = 85 (33.6%)	14/168 (8.3%)	22/85 (25.9%)	0.001
	91.4 (81.1, 102.8)	149.3 (119.1, 187.1)	
TDF N = 73 (28.9%)	33/180 (18.3%)	3/73 (4.1%)	0.003
	111.4 (95.7, 127.6)	99.0 (78.0, 122.5)	
Diabetes N = 24 (9.5%)	34/229 (14.9%)	2/24 (8.3%)	0.385
	107.6 (95.7, 121.1)	108.6 (71.8, 164.4)	
CKD N = 14 (5.5%)	28/239 (11.7%)	8/14 (57.1%)	0.001
	99.1 (88.9, 110.2)	455.0 (291.7, 707.9)	
**Admission diagnoses**			
Pneumonia N = 43 (17.0%)	30/210 (14.3%)	6/43 (14.0%)	0.955
	108.6 (95.5, 123.3)	103.0 (79.6, 133.7)	
CCF N = 39 (15.4%)	27/214 (12.6%)	9/39 (23.1%)	0.085
	105.9 (93.5, 120.0)	118.0 (88.7, 156.7)	
Gastroenteritis N = 30 (11.9%)	36/223 (16.1%)	0/30	NA
	105.7 (93.7, 119.1)	123.9 (88.9, 172.6)	
Tuberculosis N = 20 (7.9%)	35/233 (15.0%)	1/20 (5%)	0. 218
	108.1 (96.0, 121.6)	104.2 (68.5, 158.1)	
Other sepsis N = 13 (5.1%)	34/240 (14.2%)	2/13 (15.4%)	0.903
	106.4 (94.8, 119.4)	134.4 (74.0, 243.8)	

TDF = Tenofovir Disoproxil Fumarate CKD = chronic kidney disease CCF = congestive cardiac failure

### Comorbidities

There were 100 PLWHIV included in this study, comprising 39.5% of the total study population. AKI was less common amongst PLWHIV; of those PLWHIV, eight patients developed AKI (8%), compared to 18.3% of patients not known to be infected (n = 153); p = 0.022. In the total study population, there were 73 (28.9%) patients receiving ART with TDF, of these patients, three (4.1%) developed AKI. AKI was seen in 33 of the 180 individuals not taking TDF (18.3%); p = 0.003. There were 85 (33.3%) patients known to have a history of hypertension in our study population. Of these, AKI was present in 22 (25.9%); 14 of 168 patients (8.3%) without a known history of hypertension developed AKI (p = 0.001). There were 24 (9.5%) patients with a known history of diabetes included in our study population, 2 (8.3%) developed AKI, whilst 34/229 (14.9%) without diabetes developed AKI. No significant difference was observed between the proportion of patients with and without diabetes who developed AKI (p = 0.385). There were 14 patients in our study known to have a prior history of CKD (5.5%). Of these individuals, eight (57.1%) developed AKI. AKI was also seen in 28/239 (11.7%) patients without a known history of CKD (p = 0.001).

### Admission diagnosis

The commonest recorded diagnoses precipitating hospital admission in this patient cohort were pneumonia (n = 43, 17%), congestive cardiac failure (n = 39, 15.4%), gastroenteritis (n = 30, 11.9%), tuberculosis (n = 20, 7.9%) and other causes of sepsis (n = 13, 5.1%). No significant differences were seen in the proportion of patients developing AKI according to these admission diagnoses. Other admission diagnoses included: hepatitis B (n = 2), malaria (n = 3), meningitis (n = 10), obstetric complications (n = 4) and use of traditional medicines (n = 2). The numbers in each category were too few for analysis in relation to AKI status.

During the duration of the study follow up, no patients received dialysis and no patients died. The number of patients included in our study who required dialysis during their admission, but after the 48-hour period of observation of our study, is not known.

### Predictors of AKI

In logistic regression analysis, AKI amongst adult patients admitted to medical wards was related negatively to female sex (OR 0.461, 95% CI 0.211, 1.003; p = 0.051), and was positively predicted by the presence of comorbid hypertension (OR 3.292, 95% CI 1.52, 7.128; p = 0.003), and a known history of CKD (OR 6.034, 95% 1.792, 20.313; p = 0.004). Age was not a significant predictor of AKI.

## Discussion

In this study of 253 adults admitted to the acute medical wards of the largest urban tertiary hospital in Zimbabwe, AKI, defined using the most recent KDIGO criteria, was common, affecting 14.2% of newly admitted patients. AKI was predicted by male sex, a history of hypertension and a history of CKD. The estimated incidence of AKI in sub-Saharan Africa varies from two percent to over twenty percent, however published series vary in their design, setting, participant characteristics and the definition of AKI used [6, 14, 18, 19]. For instance, a retrospective study conducted in a rural Ethiopian hospital applied the Acute Kidney Injury Network (AKIN) definition of AKI [20] to a cohort of inpatients who had their serum creatinine measured during routine clinical care and found an incidence of AKI amongst medical patients of 20% [18]. A retrospective study of 6,151 medical admissions to a Nigerian teaching hospital applied the RIFLE criteria [21] and found an incidence of AKI of 1.9% [19]. In a prospective study conducted in a large hospital in Malawi, 892 medical patients were followed from the time of hospital admission until discharge, and all patients had serum creatinine measured regularly. The incidence of AKI was 17.7%; a rate similar to that which we report here [14]. The rate of AKI we report here is lower than that reported in hospitalised adults in high-income countries, where the pooled incidence rate of AKI is 22% [6]. In Africa, AKI is predominantly a community-based illness and is seen in patients who are young, with a single aetiology [3]. Community-acquired AKI is challenging to quantify and different approaches to capture these cases have been used [22]. The methodology used in our study focused on hospital inpatients developing KDIGO-defined AKI within the first 48 hours of admission to the medical wards and was not designed to detect community-based AKI.

Our study highlights some inherent limitations of the KDIGO definition of AKI when applied in resource-limited settings, where baseline creatinine is frequently unknown and CKD is prevalent, but often undiagnosed. In our study, 93 patients (36.8%) were found to have an elevated creatinine, but only 36 met the KDIGO definition of AKI, with an increase in serum creatinine of ≥26.5μmol/L over 48 hours. Among the 57 patients with an elevated serum creatinine, but not meeting the KDIGO definition of AKI, 14 reported a history of chronic kidney disease. The remaining 43 patients may have had either undiagnosed CKD, or community acquired AKI not captured by the KDIGO definition, or both. In our analysis these patients were considered as not meeting criteria for the diagnosis of AKI. A recent study concerning the global prevalence of AKI used a modified, broader, definition of AKI. This was defined as an increase or decrease in serum creatinine of greater than or equal to 26.5μmol/L, or an increase of 50% or more within three days of a known index baseline value [22]. Using this modified KDIGO definition of AKI, 99 patients (36%) in our study would be defined as having AKI. There is a concern that this approach may include patients without true AKI, for instance those with unknown, pre-existing, CKD who may experience a fall in serum creatinine greater than 26.5μmol/L from a higher baseline value in the absence of true AKI [22]. Of note, all patients with a previous history of CKD included in our study met this broader definition of AKI. The lack of any baseline serum creatinine measurements in our patient cohort limit the applicability of components of the KDIGO definition of AKI but is an accurate reflection of patient characteristics in some resource limited settings.

Whether the pattern of hospital-acquired AKI occurring in large hospitals in low-income countries is similar to that in high-income countries is unclear [23]. AKI occurs secondary to a broad range of aetiologies in low-income settings including sepsis, hypovolaemia, infectious diseases (in particular gastroenteritis), haemolytic uraemic syndrome and post-infectious glomerulonephritis [23]. HIV infection has been associated with AKI, generally in the context of sepsis and with the use of nephrotoxic anti-retroviral drugs including TDF [23]. In a large prospective study of medical inpatients in Malawi, an admission diagnosis of gastroenteritis, acute tuberculosis and liver failure were associated with AKI [14]. In our study, admission diagnosis was not a predictor of KDIGO-defined AKI. In addition, a history of HIV infection did not predict AKI and a history of the use of TDF-containing ART was associated with a lower frequency of AKI. In our study an admission diagnosis of gastroenteritis and the presence of anaemia did predict a *fall in creatinine* of greater than 26.5μmol/L at 48 hours; one explanation for this observation, and the difference in the observed associations between our study and Evans et al. in Malawi, is that these patients had suffered community-acquired kidney injury that recovered during the study follow-up period, but were not captured in the AKI cohort of our analysis.

Longer-term outcomes of AKI were also not examined in our study; high patient mortality and poor access to dialysis have been described previously [15]. AKI severe enough to require dialysis is common and mortality is reported to be as high as 44% [14]. The absence of patient mortality observed in our study may be explained by our inability to obtain informed consent from the most acutely unwell admissions during the period of study recruitment. As well, the absence of acute dialysis observed may be explained by logistical challenges in organising renal replacement therapy at our centre; it is unusual to be able to initiate acute dialysis until at least 24–48 hours after admission.

In our study, we demonstrate that male sex, a history of hypertension and CKD were significant predictors of AKI. Hypertension is a recognised risk factor for AKI among African patients [9], although this is not a consistent finding [24]. Comorbidities, such as hypertension, are increasingly prevalent in sub-Saharan Africa [25]. There was a known history of CKD in 5.5% of patients in our study; none had a known baseline renal function result available at the time of their admission. This rate is lower than suggested by a recent meta-analysis in sub-Saharan Africa, which reported a prevalence of CKD in 13.9% [26]. The lack of a known result from previous measurements of renal function, if performed, likely led to an underestimate of the true prevalence of CKD in our patient cohort. An association between male sex and AKI in sub-Saharan Africa has been previously reported [15]. Male patients predominate in other series of AKI in sub-Saharan Africa; an observation previously attributed to the relative disempowerment of women when accessing healthcare in this setting [15]. Female patients were not under-represented in our study cohort; accounting for 53.7% of patients. A biological or environmental susceptibility among males to AKI is an alternative explanation, although, it remains to be defined [15].

This study had a number of limitations. The sample size was not sufficiently powered to confirm or refute previously described associations between admission diagnosis, comorbidities and AKI. The short period of follow up meant that some cases of AKI developing after this time-point may have been missed.

In this study we demonstrate a high prevalence of AKI in newly admitted medical patients in large hospital in urban Harare, Zimbabwe. Risk factors for AKI were common, including hypertension, CKD and HIV infection. Patients with pre-existing risk factors, particularly CKD and hypertension, were most likely to develop AKI. The strict application of KDIGO criteria for AKI in newly admitted medical patients is confounded by patients with resolving community-acquired AKI and/or undiagnosed underlying CKD without known baseline serum creatinine measurements.

## Supporting information

S1 Data (XLSX)
